# ‘None Regardless of Reputation Will Be Received’: Midwifery and Commercial Bodywork in Urban Scotland *c*. 1780–*c*. 1840^1^

**DOI:** 10.1093/shm/hkaf002

**Published:** 2025-02-25

**Authors:** Eliska Bujokova

**Affiliations:** University of Glasgow, Centre for Gender History, University Gardens, Glasgow G12 8QH, UK

**Keywords:** midwifery, care entrepreneurship, care sector, bodywork, eighteenth century, nineteenth century, women’s work, urban Scotland

## Abstract

This article presents two case studies of Scottish midwives, Mrs Laidlaw from Edinburgh and Mrs Alexander from Aberdeen who used newspaper advertising to promote their establishments. Primarily providing for women wishing to conceal their pregnancies and find alternative provisions for their children, the two providers marketed the discreet nature of their practice. Together their stories contradict the dominant strands of historiography on early nineteenth-century midwifery focussed either on its increasingly professionalised and masculinised nature or its rootedness in community practice, largely resistant to commodification. Instead, this article centres on female care and bodyworkers who found opportunities for entrepreneurship in the commercialised care sector. Through focussing on the services offered and their clandestine nature, it elucidates the experiences of lying-in of unmarried mothers of means. Highlighting the midwives’ ability to adapt to the socio-cultural fabric of motherhood, it contributes to the histories of female entrepreneurship and its many forms within the care sector.

## Notice to the Public.

**RETIREMENT FOR PREGNANT LADIES.**

And others in Ordinary Life, at No 8, **SUMMERHALL**, opposite the South East Entry to the Meadow Walk, Edinburgh.

**MRS LAIDLAW, MIDWIFE**, at her Furnished Lodgings. No. 8, Summerhall, takes the methods of informing the Public, that she continues to **BOARD** and **LODGE** such **PREGNANT FEMALES** whose situation requires a few weeks retirement, with her aid; but none regardless of reputation will be received.

Mrs Laidlaw provides Milk and Dry Nurses in town or country for the children, if required. She feels grateful to the Nobility, Gentry and others, who have favoured her with their support above forty years in Edinburgh. She hopes they will continue their confidence in her, which she will endeavour to merit.

Address to Mrs Laidlaw, Midwife, No. 8. Summerhall, Edinburgh, where paid letters will meet attention.

N.B.-Mrs Laidlaw’s Lodging’s are self-contained, with a garden, with iron railings in front.

May 10. 1832.^2^

Medical advertising was common in Victorian Scotland, and many practitioners chose the medium to publicise their services, remedies and techniques to potential clients. By the late eighteenth century, Edinburgh had become a medical metropolis with large numbers of practitioners offering their services through various channels. Many women found employment in the growing care sector as midwives, nurses and childcare providers, and various bodyworkers that fell in between neat occupational denominators. Advertisements by midwives such as the one above placed by Mrs Laidlaw were rare, however, with most women in the profession practising within their communities, relying on thick forms of trust rather than the anonymity of popular press.^3^ In this case, it was the promise of anonymity that brought pregnant women to practitioners such as Mrs Laidlaw, with the hope of concealing a pregnancy and preserving one’s marriage or reputation. Mrs Laidlaw was the first midwife using the medium of newspapers to expand her clientele detectable in the Scottish press, capitalising thus on the willingness of some women to pay a premium for the discreet nature of her services. Through further research, I discovered over 70 of her adverts across Scottish and English titles placed between 1801 and 1837, which enabled me to trace her life and career with an unusual level of detail. In this article, I place her story alongside another Scottish midwife who chose newspapers for publicity, Mrs Alexander residing in Aberdeen. Apart from these two women, I found no other midwife in Scotland that advertised their services in the newspapers, making the two women rather unusual. Illegitimacy rates were high across Scotland, although subject to regional variation, and rose especially in the late eighteenth and early nineteenth centuries, a period of significant socio-economic change. Clandestine births were not a novelty and the services offered by Mrs Laidlaw and Mrs Alexander were likely offered by other practitioners.^4^ The openness of the adverts placed by the two women, however, represents a unique case of commercialising the practice whilst building on the expansion of travel for the purposes of receiving medical care, especially prominent in Edinburgh.^5^ Whilst many male practitioners adapted their marketing strategies to the advantages of the press, female commercial bodyworkers utilising the medium were far fewer, making the two women unusual.

Whilst the concept of bodywork is well established across the social sciences, it is less frequently used by historians. Increasingly, historians have been turning their attention to the labours of tending the bodies of the young, aged and infirm outside of the medical sphere.^6^ More work still remains to be done on those aspects of bodywork that do not involve relations of physical dependency resulting from infirmity or age, including the work of servants and landladies. As Mary Fissell argues, broadening the focus of medical histories to encompass varied forms of bodywork is key to grasping the historical realities of caring.^7^ This article contributes to this quest through adding evidence of commercial midwifery. In this article, I use the definition of bodywork put forth by Twigg et al. as the ‘work that focuses directly on the bodies of others: assessing, diagnosing, handling, treating, manipulating and monitoring bodies that thus become the object of the worker’s labour’.^8^ Crucially, Twigg shows how the understanding of the ‘work undertaken on the bodies of others as “body work” provides a mechanism for relating work in the sphere of health and social care to that in other sectors, opening up new avenues for research’.^9^ When researching care in the past, this reality is ever more pressing, given the lack of clearly demarcated occupational categories and the fluid nature of work. Applying the concept of bodywork onto midwifery proves useful in allowing for the inclusion of the varied practices linked to the care of the mother and child pre- and post-birth for a prolonged period of time. As will be shown, both Mrs Laidlaw and Mrs Alexander provided and facilitated a range of short- and long-term forms of bodywork and simply focussing on their involvement in assisting in childbirth would be inadequate. By instead centering the practice of bodywork in its variedness, we can begin to grasp the range of services they offered in their interlink, placing them firmly within the socio-economic and cultural contexts of urban Scotland in the late eighteenth and early nineteenth centuries. Barbara Mortimer’s work on domiciliary nurses in mid-nineteenth century Edinburgh begins to demonstrate the numerous avenues for female care entrepreneurship, but also the interrelatedness of varied caring tasks carried out by individuals with diverse occupational roles. Private nurses as well as midwives feature as employers of numerous other practitioners, highlighting that many women in the care sector ran independent businesses and gained strong occupational identities, contrary to the enduring assumption of caring as a low paid and low status occupation.^10^

Much has been written about the changing nature of medical and midwifery provision in the context of increased industrialisation and urbanisation.^11^ Historiography on midwifery in the eighteenth and nineteenth centuries centres on its assumed transition from a feminised community-based practice rooted in the innate and embodied understanding of pregnancy and childbirth to an increasingly medicalised sub-field of general medicine, practised largely by male physicians, relegating female midwives to community practice, in spite of the continued involvement of female midwives in the vast majority of childbirths.^12^ Through its expansion within the institutionalised space of the university and the infirmary, midwifery is set alongside the growing compartmentalisation of medicine and care and the subsequent establishment of specialised institutions, in this case the lying-in and maternity hospitals. The history of midwifery in the eighteenth and nineteenth centuries thus largely exists between two demarcated scholarships, the first focussing on the growing field of obstetrics and male medical practice, and the second on female, community-based and less commodified practice.^13^ Whilst the stereotypes of the untrained and ignorant Sarah Gamp have rightly been abandoned by historians, the historiography of commercial midwifery in the eighteenth and nineteenth centuries remains to be written.^14^ Expanding on Mortimer’s work, this article looks at the practice of midwifery in the context of care entrepreneurship, as overlapping with other forms of care, medicine and service work. It provides evidence of the continued engagement of women in midwifery beyond community practice. Apart from their shared use of the press, the lives and careers of Mrs Laidlaw and Mrs Alexander had little in common, and their material circumstances differed greatly. Whist Mrs Laidlaw practiced independently throughout her life and built a highly profitable establishment, Mrs Alexander’s life-course followed a path of instability, marital separation and downward social mobility. This article employs their varied stories as a way of accessing the histories of paid female care providers in Victorian Scotland. It contributes to the historiographies on late-eighteenth and early-nineteenth century midwifery and commercial bodywork in the urban space. More broadly, it speaks to the growing scholarship on women’s entrepreneurship, highlighting the varied avenues women took to make a living, gain economic independence and run successful commercial ventures.

Placed within the broader context of burgeoning of private practice on the one hand and institutional expansion and standardisation of care provision on the other, the two midwives practised in the increasingly anonymised urban care sector, both drawing on alternative ways of soliciting clients or devising strategies in which the discretion of their services proved advantageous. Crucially, this article thus sits within the broader context of the expanding commercial care sector, populated by regular and irregular practitioners, care and childcare providers and various bodyworkers making a living through care labour.^15^ As shown by Mary Fissell, the world of health and healing was increasingly interconnected with the market of print, with medical books and tracts seen as an extension of practice.^16^ Advertisements placed by practitioners were integral to the expansion of medical print and thus reveal the relationship between the two sectors. Notwithstanding growing access to, and consumption of the public press in Scotland, surprisingly little has been written on its role in the proliferation of medical knowledge and practice, largely owing to the fact that Scottish cities never reached the level of commercialisation and anonymisation of market exchange pictured by the London-based scholarship of the medical marketplace.^17^ As demonstrated by Helen Dingwall, however, popular press and medical advertising played a key role in the intellectual, institutional and cultural contexts of Edinburgh, shaping the commercialised practices of medicine within and without the walls of the city’s many medical institutions. As shown by the examples of Mrs Laidlaw and Mrs Alexander, medical advertising was closely intertwined with the broader market for care and bodywork, childcare, domestic service and the expanding service sector more broadly. Both midwives provided a range of services, employed servants and procured nurses and servants for their clients. It is by placing their work as midwives alongside that performed by wet, dry and sick-nurses, body servants and landladies that the broader care sector emerges.

## Helen Laidlaw, the Edinburgh Midwife

**TO THE PUBLIC.**

**MRS LAIDLAW, MIDWIFE**, Clamshell Land near the Cross, Edinburgh, takes this method of informing the Public, That she continues to receive PREGNANT WOMEN as Boarders or Lodgers, whose situation requires secrecy, if they have letters from some person of respectability, or are recommended, as none regardless of reputation will be received.- Mrs Laidlaw provides Nurses in Town or Country for the Children; or, if more agreeable to those concerned, they may be relieved of the Child altogether, on paying a sum for that purpose. As Mrs Laidlaw has practised nineteen years in Edinburgh, she hopes for the continuance of the confidence the Public has been pleased to confer upon her.- Address for Mrs Laidlaw, midwife. No. 9, south side High Street, Edinburgh, where post-paid letters will be attended to.

Edinburgh, Sept. 7. 1805.

- Nurses and Servants provided with places as usual.^18^

Mrs Helen Laidlaw started practising midwifery around 1784. We know very little about her until then, though the historical record of her life and career becomes surprisingly rich thereafter. She practised the profession until her death in 1837, as suggested by her numerous adverts and listings in the Post Office Directories, a subscription list where Edinburgh residents shared their personal and business details for the purposes of advertising. She advertised widely across the Scottish and English press, most frequently appearing in London, Newcastle, Cumbernauld, Edinburgh, Inverness, Aberdeen and Perth to ‘pregnant ladies’ who travelled from afar, wanting to return unencumbered by their newborn children. Her services included paying ‘the utmost attention to such Ladies as are properly recommended and with retirement’ whilst also keeping a ‘Register Office for Nurses and Servants who can procure proper Certificates of their Character’, furnishing ‘nurses for lying-in Ladies’ and wet and dry nurses in town or country as well as children’s clothing.^19^ For mothers hoping to find alternative provision for their newborns, she proposed that they ‘may be relieved of the Child altogether, on paying a sum for that purpose’.^20^ Additionally, she promised that the ‘greatest secrecy can be depended on whenever she is entrusted with a person whose case requires it’.^21^ Her broad range of services reflected the complex needs of pregnant women in varied circumstances, seeking subsequent care and childcare provision, undoubtedly earning Mrs Laidlaw a premium for securing all that was needed at once. Her establishment catered to a broad clientele, drawing on the well-established practices of medical advertising, actively stepping outside the personal networks customarily inhabited by midwives. Hers was a different type of service, catering to women with means though ones unable or unwilling to draw on community based provision, counting unmarried mothers, women who maintained extramarital relations and others who simply wanted to escape the ties of motherhood.

Practitioners such as Mrs Laidlaw placed themselves within the moral economy that regulated the experience of pregnancy and motherhood that differed greatly for those legally wed and those who conceived outside of wedlock. She was able to monetise the unwanted pregnancies of her clients, with a range of services offered to those hoping to give birth in anonymity and leave unencumbered by their unwanted children. As Jennifer Johnson-Hanks demonstrates, motherhood can be viewed as socially contingent as opposed to gestational, a role socially and culturally assumed as opposed to realised through the physical act of giving birth.^22^ Examples of the variedness of care arrangements for unwanted children for the better off highlight the element of choice available to mothers with access to cash contrasted by the choicelessness of motherhood experienced by poor women, who had few options beyond abandonment.^23^ Historians such as Laura Gowing and Patricia Crawford brought forth rich evidence of the experiences of poor unmarried mothers, many of whom were met with hostility, in extreme cases resulting in parishes removing women in labour to avoid financial responsibility for them and their children.^24^ Equally, Katie Barclay and Kate Gibson amongst others shed light on the elites who had options available to them when dealing with adultery and illegitimacy.^25^ However, experiences of middling women remain overshadowed by archival silences and prescriptive cultures of sexual morality, with less direct evidence available. As Angela Joy Muir suggests, the experiences of single pregnant women were extremely varied, and this was largely determined by their socio-economic status.^26^ Practitioners such as Mrs Laidlaw and Mrs Alexander catered to middling women wishing to conceal a pregnancy and seeking alternative provision for their children, offering solutions that were within reach financially and did not require an expensive sojourn abroad.

The first mentions of Mrs Laidlaw appear alongside another midwife, also called Mrs Laidlaw both advertising their services in the Post Office Directories. One of the two midwives resided in Burnett’s Close and the other in the Head of Horse Wynd, both in the Edinburgh Old Town.^27^ A Scottish midwife named Mrs Laidlaw also appeared in obituaries published in the *London Star* in February 1814 and the *Sun* in March 1814, amongst other newspapers. The obituaries commemorated her as living in the village of Sanquhar in Dumfriesshire and assisting in over 1400 childbirths in over 50 years of her practice.^28^ According to the information from these obituaries, this Mrs Laidlaw trained in midwifery in Edinburgh and was certified in 1761. She practised in the village until the age of 86 and died aged 88, in January 1814.^29^ Described as a ‘respectable widow’, it is possible that she was also a mother, that is, the mother of Helen, who was 20 years her junior and possibly brought into the profession by her mother, then also living in Edinburgh, though this information remains speculative. The two women lived and practised in Edinburgh in the 1780s and 90s, after which period only one midwife named Mrs Laidlaw remains in the directories, possibly due to Mrs Laidlaw senior’s removal to Sanquhar. Whether Helen Laidlaw inherited her trade from her mother or not, she began practising around the age of 34, roughly the same age as Mrs Laidlaw senior 20 years earlier, and worked in Edinburgh for 53 years. Unlike Mrs Laidlaw senior who was described as a widow, Helen Laildlaw’s marital status was never mentioned and the occupational descriptor ‘midwife’ was the only identifier used.^30^ The absence of a mention of a husband or her status as a widow in any of her numerous appearances in the court of law suggests that she was unmarried from at least 1801, though she may have been separated at an earlier stage. No evidence of her having living children exists. Single women engaged as midwives were not the norm, with the expectation being that midwives had a first-hand experience of childbirth, as well as gaining access to reproductive knowledge through marriage.^31^ In the context of Edinburgh, however, many single women were employed as care providers, especially in the expanding institutional context, and had access to midwifery training offered by the Edinburgh Infirmary. Their role as skilled practitioners was more readily recognised than that of women working in community settings, who gained their skills through practice in the locality. Working in the capital throughout her career, Mrs Laidlaw never served as a community wise-woman and was able to build her establishment as a single woman.

Mrs Laidlaw continued to advertise in the directories, and from 1801, also in the British press. Her commitment to self-promotion as well as her predilection for litigation provide a clear trajectory of her business ventures, which alongside midwifery and childcare-related services, included shop keeping, liquor dealing, and real estate. The size and scope of her establishment offer an example of a private proto-clinic that combined the provision of home-based care with a larger scale establishment, which, through the employment of a number of staff and gradual spatial expansion, enabled Mrs Laidlaw to diversify her services and make considerable profit. Her business model, which combined private domestic provision with an institutional larger-scale model, demonstrates her ability to combine traditional practice with the increasingly centralising, institutionalising model of care provision, symptomatic of the expansion of the public care sector. Establishments such as this would have been unavailable to middling women prior to the eighteenth century, which saw the diversification of commercial care.^32^ Mirroring the new formats of care provision, entrepreneurs such as Mrs Laidlaw adapted their businesses to fit the growing public demand and confidence in institutional authorities whilst remaining rooted within the moral economy operating on the principles of trust, recommendations and elite endorsement, as reflected in the language of her adverts. Crucially, her strategy to apply the principle of economy of scale onto the provision of care through taking in multiple clients at once enabled her to minimise the cost of staffing and provisions, an approach followed by emerging care institutions, enhanced further by running the establishment alongside her household.

At the start of her career, Mrs Laidlaw lived and worked in various rented premises in the Edinburgh Old Town. In 1800, she moved to the convenient location in close proximity of the Infirmary, No. 1 Infirmary Street, where she leased a three-bedroom property with a kitchen and a front shop at £16 per annum.^33^ The area around the Edinburgh infirmary was populated by clusters of care and lodging providers, echoing the professional networks that formed between medical men associated with the hospital and private carers who often relied on their contacts. Midwives such as Helen Laidlaw were involved in such networks liaising with medical men, domiciliary nurses as well as casual care providers and domestic workers. Running a register office, she kept a list of servants, childcare providers and sick-nurses desiring employment, who paid a fee to have their name entered in order to be contacted by potential employers. Register offices were a common way to seek employment for service providers, and Edinburgh business owners with vast social networks were ready to use the opportunity to gain income.^34^ Additionally, medical practitioners often kept registers of care and bodyworkers they could call on in need of nursing assistance.^35^ Mrs Laidlaw kept up the register office throughout the years of her practice, demonstrating her continued place in the local networks of trust alongside primarily providing for out of town clientele.

Around 1800, she rented a small shop nearby from Colin Lauder Esq, where she set herself up as a grocer and an ale vendor next to a woollen drapers’ who subsequently brought her to court for damaging their shop front.^36^ In this first recorded instance of her presence in the court of law, she presented herself as a midwife and a solvent citizen of Edinburgh, demonstrating a strong occupational identity derived from her work in the care sector.^37^ Subsequently, she continued her other ventures in the service sector, abandoning her vending business as her midwifery practice grew in profitability. In February of the following year, she placed her first detectable advert in the London-based *Morning Chronicle*:^38^

**TO PREGNANT LADIES.**

**MRS. LAIDLAW**, Midwife, Edinburgh, takes this opportunity of acquainting them and the Public that she continues to keep a Register Office for Nurses and Servants who can procure proper Certificates of their Character. Mrs. Laidlaw continues to pay the utmost attention to such Ladies as are properly recommended and with retirement, as no other will be received. The greatest secrecy may be depended on whenever she is intrusted with a person whose case requires it. Nurses provided also for Lying-in Ladies.

Mrs. Laidlaw’s having practiced with success for fourteen years in Edinburgh is a sufficient testimony of her abilities. She continues to let Lodgings to such Ladies only, at her House, No. 1, Infirmary-street, opposite the New College, Edinburgh.

Letters, post paid, duly attended to.^39^

Her decision to advertise in London prior to Edinburgh reflects the wider circulation of London based papers, reaching readership across Britain. Whilst in the Scottish press Mrs Laidlaw was unique, many midwives advertised their services in London-based titles, reflecting the wider pool of commercial practitioners in the capital.^40^ Additionally, however, it shows her orientation towards a clientele further afield coming to Edinburgh to conceal a pregnancy and potentially leave their often-illegitimate children behind, temporarily or *altogether* as her numerous adverts proposed.^41^

By 1803, Helen Laidlaw moved to her ‘airy house’ in Bristol Port, near the Meadow Walk, where she only stayed for 2 years. By 1805, she purchased a five-bedroom apartment with a kitchen and a shop on Clamshell Land, a three-storey townhouse located at No. 150 High Street, where she lived until 1816.^42^ During this time, she also purchased a three-room house with a front shop on No. 3 Tobago Street valued at £105 in 1813, where she initially ran her registry office and later rented out the property at £12 per annum.^43^ Her Tobago Street property brought her to court again in a suit against a potential buyer, John Aldie, who had a change of heart and refused to purchase the property after closing the deal. The midwife won the case and damages of £25 in addition to Aldie agreeing to purchase the property at the original price.^44^ Residing now in the house on the Royal Mile, the main street in the Old Town, she continued to ‘receive pregnant women as Boarders or Lodgers, whose situation requires a few months or weeks retirement’.^45^ With the vision to relocate to a ‘self-contained house in the vicinity of Edinburgh, or within a few miles, with a garden or a piece of ground’, she began to advertise for a new property. In 1817, she moved to her new premises on No. 8 Summerhall, where she remained until her death in 1837. The new house was ‘self-contained within a Garden, and Iron Railing in front’, furnished with a cold-water bath available to residents as well as paying customers at the cost of 1 day per visit.^46^

As reflected by the premises she occupied, Mrs Laidlaw’s establishment grew considerably in size over the 50 years of her practice. Such expansion would have been matched by the number of staff she employed, consisting of domestic servants, nurses and wet-nurses. With the purchase of the Summerhall property, she would have added to her staff a groundskeeper or a gardener, also in charge of the public baths. Her long-term arrangements for her patients and her Clamshell and Summerhall properties containing a kitchen suggest that she employed a cook charged with the preparation of food and medicinal remedies. Lastly, her maintenance of several properties at once, running her clinic, a separate shop and a registry office, required her to delegate a considerable amount of her responsibilities, hiring a clerk and a shopkeeper. Running her clinic until the age of 87, she possibly took the role of an overseer or a domestic manager, delegating much of the physical labour involved to others. Whilst detailed evidence of her staffing does not survive, the spaces she occupied provide a lead from which some of her household composition can be inferred. Her role as an employer is crucial here, echoing Mortimer’s work on private nurses and highlighting the variability of care and bodywork in the Scottish capital.^47^

Mrs Laidlaw’s adverts varied greatly over the 36 years of her advertising, though the double emphasis on respectability and secrecy was made repeatedly. Examining their content, language and services offered alongside the place of publication of the newspaper in which the adverts were placed reveals a lot about the potential clientele and the way in which the midwife diversified her services and subsequently marketed them to a varied audience. Her early adverts placed in London and Newcastle titles emphasised flexibility and discretion alongside the great variety of services offered. Her 1803 advert in the *Newcastle Courant* highlighted that she ‘continues to let Lodgings, and practise Midwifery, *and particularly to receive lying-in Women who wish Concealment,* if they have any able Person to recommend them, as none else will be treated with’ (original emphasis). She also offered to ‘attend Ladies in their own Houses and provide Milk or Night Nurses. Children put out to Board in Town or Country; or if more agreeable to those concerned, a Sum of Money will be taken to relieve the Parents altogether’.^48^ This advert was the only one offering pregnancy care in women’s own homes, and Mrs Laidlaw likely abandoned the practice as her clientele grew and she obtained larger premises. With her orientation towards out-of-town clients, her focus thus remained on providing care within her own establishment. Whilst most childbirths took place in the mothers’ homes, Mrs Laidlaw’s practice appears unusual, suggesting that the women who sought her aid were not local, but rather ones relying on her pledge of discretion, probably for an enhanced fee.

The emphasis on respectability, made in all of Mrs Laidlaw’s adverts functioned as a replacement of the trust afforded by acquaintance and neighbourliness, a warranty that the client would not run off without settling their expenses or abandon an unwanted child. As shown by the 1807 example from the *Caledonian Mercury*, one of her first Edinburgh adverts, respectability was tangible, not a marker of virtue or status, but rather a proof of solvency. Mrs Laidlaw requested that her lodgers ‘either bring letters from some person of respectability, or deposit a sum of money for the inlying, and other expenses, then it will not be required to know the parties’.^49^ With respectability functioning as a linguistic code for solvency, Mrs Laidlaw’s messaging was clear, and she was to provide for anyone able to pay, with abundant domestic comforts, additional services and few questions asked. Interestingly, her later adverts assumed a rather more coded form, with fewer promises of secrecy and care offered to ‘pregnant ladies’ as well as ‘others in ordinary life, whose situation requires a few weeks or months retirement, with her aid’.^50^ Having become more established, she likely needed less explicit marketing of the services she offered. Her long years of practice as well as profits made manifest through her purchases of real estate testify to the success of her private establishment, with her continued presence in the popular press serving as a reminder to her clientele, though becoming less detailed in describing the services offered. Her expansion to care for people in need of nursing or convalescent care as opposed to only catering to pregnant women shows again her ability to adapt to the Edinburgh medical market, but also the capacity of her establishment and its staff to provide varied care. Whilst continuing to care for those whose ‘respectability’ needed to be proven, it appears that her own never suffered. Her presence in the press, as well as her appearances in the court of law, betray her strong professional identity, economic security and access to patronage of the city’s elites. Whether the nature of her establishment was ever contested by municipal authorities or the kirk is difficult to ascertain, though her continued business success demonstrates her ability to resist any such potential barriers.

None of the midwife’s adverts hint at the vending of abortifacients, commonly advertised as ‘female’ or ‘obstruction pills’. The nature of her establishment, however, hints at the likelihood of her assisting in pregnancy termination alongside the services offered in the press. Her omission to advertise abortifacients and contraceptives is curious, especially in her early adverts, perhaps testifying to the more implicit mode of advertising in the Scottish press, where no such adverts appear prior to 1800 and remain less common than in England thereafter.

Whilst her adverts habitually closed on her expression of gratitude to the ‘Nobility, Gentry, and others who have favoured her with their support’, her clientele was unlikely to have counted many members of the landed elites.^51^ Rather, as her 1817 advert in the *Mercury* suggested, ‘those of respectability, although not in high life’ were those seeking her treatment. One such client was Isabella Blyth, the unmarried daughter of Swan Blyth, a ship captain resident in Leith. The 20-year-old lodged with Mrs Laidlaw in 1808, during the period of her lying-in, when her father would habitually visit and dine with the two women. Isabella’s mother and sister Ann were not mentioned and her father was the only family member to appear in the record. This rather mundane case was only brought to the archive as Swan failed to settle his debt of £10 Scots (about £1 Sterling) to Mrs Laidlaw, only part of the amount charged for Isabella’s care.^52^ It is unclear how much the total bill amounted to, however, with midwives catering to poor women habitually receiving a fee ranging between 2 s and 5 s, Mrs Laidlaw’s fees were out of reach of those without means. As Isabella, the daughter of a naval officer, is the only named client of the midwife, it is difficult to ascertain who were the women Mrs Laidlaw cared for. It, however, suggests that the midwife’s assistance was unavailable to those below the middling sorts. The absence of Isabella’s child’s father and her living with her father shows a little of the lived realities of the midwife’s clients, many of whom conceived outside of wedlock and relied on her discretion. The quiet domestic scene of Swan, Isabella and Helen at dinner together point to the less punitive atmosphere faced by unwed mothers with access to cash and familial support. Isabella married 5 years later and likely bore other children to her husband John, whilst her previous pregnancy may have gone unmentioned. When money was available, the repercussions of illegitimacy may not have been felt as deeply.^53^

Many of the midwife’s adverts drew attention to the accessibility of Summerhall, being ‘only fifteen-minutes walk from Prince’s Street, where the mails arrive and depart, but Coaches are waiting to conwey passengers to their destinations, and for a trifle will set them down here’.^54^ Adverts placed in the *Perthshire Courier* highlighted that ‘the Carlisle, Dumfries, Kelso, Dunse, Jedburgh, Hawick, Dalkeith, Lasswade, and Peebles Coaches pass near the house daily’.^55^ Or, in the *Cumbernauld Packet*, she suggested that ‘many of the Mails from the South daily pass the Back of Mrs L.’s House through Clerk Street’.^56^ Mrs Laidlaw’s travel recommendations testify to the ways through which women were expected to arrive, betraying the geographic variedness of her clientele and highlighting the accessibility of coach travel to those lacking access to personal means of transport, travelling alone, in anonymity, for a trifle. Although not much is known about the actual women who came to lodge or give birth in Mrs Laidlaw’s establishment, they were likely women of means, albeit limited, often unmarried, travelling to receive care in their lying-in and hoping to provide for their new-born children at a nurse, which would enable them to travel back to where they came from, unencumbered.

Mrs Laidlaw’s services were shaped by the fact that some women had the choice to put off social motherhood despite having experienced the physical process of gestation and childbirth, relying on the midwife’s ability to conceal their condition. Many of her clients possibly had other children at a later stage, who, not born outside of wedlock, were brought up by their biological mothers. The care work provided by the midwife was contextualised by the moral economy of family formation, shaped by socio-cultural and material circumstances rather than biological ones. Crucially, Helen Laidlaw was able to monetise the normative framework that shaped her profession, developing a business strategy that enabled the mothers of unwanted children to buy loopholes into the social fabric of respectability. As Tania McIntosh has shown, nineteenth century midwives were by no means a homogeneous group.^57^ Whilst midwives practicing within their communities, facilitating the traditional rites of childbirth have been richly documented, those women who practiced midwifery as a commercial venture remain understudied. Providing primarily for women who chose to give birth outside of their communities or desiring to conceal their pregnancies, Mrs Laidlaw found a reliable market for her services.^58^ The juxtaposition between the secrecy around those she cared for and her own visibility is striking. It is also a crucial reminder that the notion of respectability was malleable and highly contingent, and for some, it served as a viable business venture.

## Ann Alexander, the Aberdeen Midwife

**LODGINGS-MIDWIFERY-NURSES.**

Mrs. ALEXANDER, MIDWIFE, No. 146 GALLOWGATE, ABERDEEN. Accommodates with Lodgings Ladies whose state of health may require retirement and attention. Mrs A. has considerable experience in the treatment of the complaints peculiar to her sex, having studied under an eminent Physician, and, after having been duly and strictly examined, obtained the certificate of the Medical Society to practise her profession.

Persons able to describe their case correctly by letter will be furnished with advice.

Careful Nurses provided for Children.

Children about two years of age received into the house, where they will be kindly treated, and have their health, education, and morals, strictly attended to. Terms from £13 to £20 per year, according to circumstances.

Persons honouring Mrs A. with their confidence, may depend on the strictest secrecy being observed; and those wishing to conceal their names, may have their Children received on certain conditions, which may be learned on applying by letter, either direct or otherwise.^59^

Unlike Mrs Laidlaw who ran her business as a sole practitioner and proprietor, Mrs Ann Alexander, née Skene, the second midwife who frequently appeared in the Scottish press, worked in partnership with her husband William.^60^ The couple first appear in 1840 in the Post Office Directories, as residing in 140 Gallowgate. William was listed as a surgeon and apothecary and Ann as a midwife. They moved to 146 Gallowgate a year later, where the pair were recorded by the 1841 census, living alongside five children bearing the last name of Alexander aged between 5 and 11, and two young women named Jane Cooper, aged 21, and Mary Wilson, aged 14, likely domestic workers, though this was unspecified in the census. By 1845, the family had moved to 144 Gallowgate, where they resided until 1848. By 1849, Ann and the family’s children left William and relocated elsewhere. William remained in this residence until 1852, living alongside a domestic servant Charlotte de Rudeval; a widow aged 24, after which he disappeared from the records.

Through the information available in her adverts, I was able to recover an outline of Ann Alexander’s life before and after her marital business. Ann Skene was born on 14th July 1798 to William and Elspet Skene in Tarland, a small village in Aberdeenshire. She married her husband William Alexander, then resident in Elgin, in 1826. By the 1830s, the couple lived in Aberdeen, where William made a living as a merchant, and had at least two children, Elizabeth and Margaret, residing with them. William trained as a surgeon, and although he did not finish his studies, he began practising in the 1840s. Not completing a medical doctorate was not a barrier to practising before the 1858 Medical Act, especially outside of Edinburgh and London. In spite of hosting one of Scotland’s prominent medical schools, Aberdeen had a smaller concentration of fully qualified medical men and simply having studied medicine would have been sufficient.^61^ After separating from her husband, Ann left Aberdeen and moved back to Tarland, where she was living in 1861 alongside a young boy John Enslie, registered as a boarder in the census. Now aged 64, Ann worked as a stocking worker and earned additional income through providing for John. Ann died aged 74 in 1870 in Aboyne, a village near Tarland, marked as a widow of William Alexander, surgeon. Unlike Mrs Laidlaw who ran her business independently, Ann’s dependency on her husband in their shared practice resulted in her experiencing downward social mobility after the pair separated. Her situation reflected the costliness of marital separation, moving and restarting a business, which proved prohibitive to her continuing her career as a healthcare practitioner at the same level as she had previously done. The cases of Mrs Laidlaw and Mrs Alexander demonstrate the varied ways in which women practitioners found opportunities in the care sector, as successful business owners, or practitioners working in more precarious ways, alongside male partners, often obscured by historical recording of the working lives of women.^62^

Apart from the Post Office Directories and adverts, no mention of Ann’s medical training or profession is made in the census or parish records, though this is by no means exceptional as demonstrated by Alison Nuttall.^63^ Owing to the irregular or casual nature of many midwives’ work as well as the general underreporting of women’s occupations, the census records often prove unreliable.^64^ The only official record that listed an occupation next to Ann’s name was the 1861 census, in which she was recorded as a widow and a household head alongside her designation as a stocking worker. The adverts placed by her and William in the newspapers are thus the only records of the couple’s joint business venture in the care sector. Consequently, nothing is known of Ann’s work in midwifery after leaving her husband. Whilst later recorded as working in domestic textile work, the question remains whether she continued her midwifery practice in more informal ways that went unrecorded, and if not, why. Unlike Helen Laidlaw who ran her largely profitable business alone and over a period of fifty years, Ann Alexander’s working life seems filled with precarity, change and dependence on her husband and business partner. Her adverts reveal the less conventional and largely commodified forms of care work, which represented only a small period of her working life, predominantly spent in the more obscure midwifery practices in a local community, or in different forms of work altogether.

Albeit not as numerous as the adverts placed by her Edinburgh counterpart, Ann Alexander’s intervention in the press proves equally rich in evidence, enabling the reconstruction of the establishment she ran alongside William. The above advert was the first to appear in the Scottish press, published in *The Scotsman* in June 1841. Between 1841 and 1844, Mrs Alexander advertised independently from her husband, choosing *The Scotsman* and London based *Sun* and *Bell’s Life in London and Sporting Chronicle*, whilst her adverts from 1846–7 include both practitioners (see example below). Like Helen Laidlaw’s, Ann Alexander’s choice of Edinburgh, and London-based publications reflects her aim to attract clients from out of town, where her promise of secrecy would be practicable. William Alexander’s attention to afflictions of ‘private nature’ advertised alongside Ann’s midwifery and his mercury-free remedies allude to his providing treatment for venereal diseases, particularly syphilis. Whilst not explicitly offered by the adverts placed by Ann or William, their joint practice likely encompassed abortion and contraception advice, offered through this medium to those who lacked access to informal channels of obtaining such services, or wishing greater secrecy ensured by travelling the distance to avoid being caught up in local gossip.

Contrastingly, adverts appearing in 1848, mostly placed in local Aberdeen press, such as the *Aberdeen Press and Journal*, *Aberdeen Herald*, but also *Perthshire Advertiser*, were by Mr Alexander only, reflecting the couple’s separation as well as William’s provision for a more local clientele, potentially leaving their joint focus on reproductive health behind. In the lack of a degree certificate or membership of a medical society, William was limited to private practice, ineligible to gain renown through holding an office in the emerging medical institutions. His adverts aimed to set him apart from ‘travelling quacks’ and vendors of ‘useless Quack Medicines’, disguising his own lack of fully achieved training or institutional affiliation, though not actually claiming he possessed either.^65^

## Private Medical Establishment.

**LODGINGS-MIDWIFERY-NURSES.**

WILLIAM ALEXANDER, SURGEON, 144, Gallowgate, Aberdeen, may be consulted in all cases of a private nature. A safe, speedy, and perfect cure warranted in a few days without Mercury or risk of exposure. Mr A.’s extensive practice has arisen from his greater anxiety to obtain credit for the speedy removal of disease, than from a desire to obtain money from the unfortunate, who are too often deprived of their means without deriving any benefit. There are various kinds of these diseases, each of which requires a method of treatment peculiar to itself; a personal visit or an accurate description in writing is therefore necessary. A certain cure in all cases warranted. PRIVATE LYING-IN INSTITUTION and NURSERY for CHILDREN, under the charge of an experienced Midwife.^66^

Unlike her husband, Ann presented herself as a trained and certified practitioner, endorsed by the Medical Society (The Royal Medical Society of Edinburgh), a renowned body whose accreditation lent her credibility. Whilst unusual in the context of the Scottish press as one of only two midwives who used the medium to such extent, in spite of the growing number of midwives practising in Aberdeen, she drew on the existing linguistic tropes utilised by a whole range of practitioners, drawing on the breadth of medical advertising. Despite Aberdeen’s population being less than half of Edinburgh, the directories show nearly as many midwives advertising via this medium, whilst the number of surgeons in the Edinburgh directories was four times higher than in Aberdeen.^67^ Whilst the directories are by no means a reliable indication of the real number of practitioners, they include the practitioners catering for the middling sorts, much like Helen Laidlaw and the Alexanders. Poorer inhabitants of both cities would rely on community practitioners and personal connections on the one hand and the institutions such as the infirmary and the workhouse on the other. The well off would draw on recommendations and private networks and the well earning practitioners catering to them did not need such means of advertising. The directories as well as newspaper adverts capture those outside these groups, the middling sorts, professionals, the socially and geographically mobile as well as those wishing to exit their social networks and their modes of surveillance.

Much like Mrs Laidlaw, Mrs Alexander provided a range of services, including care during and after childbirth, procuring nurses, as well as looking after children for an annual fee. Her promise of discretion echoes Mrs Laidlaw’s advertising practices, suggesting the two women ran similar businesses in the two cities, only a few years apart. Aberdeen, a much smaller and more provincial city than Edinburgh was not a destination for medical travel, though its healthcare and institutional infrastructure was fast developing, with the Infirmary established in 1739, a Lunatic Asylum in 1800 and a poor house in the 1840s. Healthcare provision for the poor was matched by numerous private practitioners, such as the Alexanders, catering to the middling sorts and the better off. Whilst traffic between London and Edinburgh was frequent, Aberdeen may have represented a more obscure location for those unable to afford a sojourn abroad to give birth to an unwanted child common amongst the wealthy, demonstrating the adaptability of the Alexanders to their geography. The sum requested for providing for unwanted children serves as an indicator of the background and status of her clientele, able to pay the fee ranging between £13 and £20, three to four times more than those who sponsored the care of children in establishments such as the Edinburgh Orphan Hospital, where children of poor parents were admitted. Crucially, as seen in the below advert placed in the *Bell’s Life in London and Sporting Chronicle* around the same time as the one above, the fee for childcare was not fixed, and in the London advert, the Alexanders’ childcare was subject to a premium, costing between £20 and £30 annually. The promise of kind treatment, healthy lifestyle, education and moral upbringing was made universally, however. Her focus in the advert on childcare provision as opposed to lying-in care is crucial in pointing toward her potential clientele who travelled to Aberdeen from as far as London for the promise of ‘the strictest secrecy being observed’ rather than the midwife’s skill or professional renown. Her offering external nurses as well as taking in her clients’ older children in a form of fostering reflect the diverse options at varied prices for those seeking private arrangements as opposed to residential institutions and orphan homes, where poverty as well as respectability of the parents were key to the children’s admission. Additionally, children placed at nurse were taken in by the Alexanders upon being weaned, mirroring the arrangements set up by foundling hospitals and other charitable institutions as well as private practitioners infamously dubbed as ‘baby farmers’. ‘Baby farmers’ were nurses who generated profit from taking in high numbers of children, many of whom suffered neglect, resulting in high death rates in such establishments.^68^ Eighteenth-century social reformers such as Jonas Hanway campaigned against ‘murderous’ and ‘mercenary’ nurses, contributing to a social panic regarding the ubiquitous practice of sending children to nurses in the country.^69^ Whilst country nursing became less popular amongst the elites, it remained widespread amongst poor families and single mothers who had few options. The panic persisted and by the 1850s, the evils of ‘baby farming’ became a much-debated subject across publications such as the *British Medical Journal* that warned against the infanticidal practice.^70^ Notwithstanding, fostering arrangements, both private and institutionally organised, remained commonplace, and in Scotland continued to be seen as superior to institutional care. Available evidence suggests that many Scottish families relied on the additional income generated through fostering, and foster children were often subsumed into families’ wider kinship groups.^71^ With the little evidence available, it is impossible to determine the nature of the Alexanders’ establishment. However, taking in older children rather than new-borns, and charging fees annually as opposed to a lump sum suggests that their establishment was making profit on the basis of long-term care arrangements, requiring them to actually provide for their charges. Notwithstanding, the context of the ‘baby farming’ panic that played out largely in the press is crucial for framing the midwives’ practices and the component of newspaper adverts. Demand for discreet lying-in services and outsourcing childcare beyond one’s immediate networks continued despite the threats to the bodies and lives of pregnant women and their children, reflecting the repercussions of women’s failure to adhere to rigid standards of sexual morality. Practitioners who specialised in such type of care provision as well as their clients had to navigate these social realities, and for some of them, these proved highly profitable.

**LODGINGS-MIDWIFERY-NURSES.-Mrs. ALEXANDER, MIDWIFE**, No. 146, Gallowgate, Aberdeen, accommodates with Lodgings Ladies whose situation may require retirement. Careful Nurses provided for Children. Children about two years of age received into the house, where they may be kindly treated, and have their health, education, and morals, strictly attended to. Terms from £20 to £30 per year. Persons honouring Mrs. A. with their confidence may depend on the strictest secrecy being observed; and those wishing to conceal their names may have their children received on lodging money sufficient to educate and bring them up in a comfortable manner.-Letters addressed as above will meet with attention.^72^

According to the 1841 census, William and Ann cohabited with five children named Elizabeth, Elspet, Margaret, Ann and Robert. Birth certificates only exist for Margaret and Elizabeth, however, with the remaining three not appearing in the records in association with Ann and William. With the knowledge of the couple’s private care arrangements and Ann’s care for John later in her life, the three remaining children were possibly fostered by the family upon the conditions highlighted by the adverts. The use of the Alexanders’ surname shows the inclusion of the foster children in the family unit. Only employing two live-in servants, the establishment run by the Alexanders was not one of scale, and the children of others were incorporated into the nuclear family. Taking in orphaned or abandoned children was a common practice of poor families; single women and widows, especially in rural areas, used to augment the household budget and in many cases provide extra hands for agricultural work. Indeed, upon moving back to her native village, Ann partook in a form of fostering organised by the parish and primarily associated with poor local widows. As recently shown by Carmen Sarasúa in the context of Spain, this practice has been largely underreported, especially in the context of families as opposed to single and widowed women, leading to the large undercounting of the contributions made by married women to household budgets through care work.^73^ Whilst no extensive body of quantitative research on the scale of this practice exists in the Scottish context, piecemeal evidence of the payments made by parishes, workhouses and charities to wet and dry nurses indicates that outsourcing childcare through a large network of ‘putting-out’ nursing arrangements was widespread across the long-eighteenth century, especially amongst poor families.^74^ In 1848, Glasgow Parochial board supported 448 children at £5 8s 4d per annum paid to the nurses hired by the parish.^75^ In Edinburgh in 1848, the parochial board paid £3 to relatives providing for children and £6 to wet nurses. This was compared with the expenditure of over £8 for the children’s maintenance in the workhouse, which was used to argue against institutionalisation of childcare by the parochial board.^76^ This was comparable with the expenses per child in the Edinburgh Orphan Hospital, a private organisation ran by a board of directors with contributions from the Society in Scotland for Propagating Christian Knowledge.

The case of the Alexanders is interesting in situating the practice largely associated with the economies of makeshift participated in by the poor within the middle class household of two medical practitioners receiving a much greater sum in return. Although an isolated case, this example proves useful in extending the practice of foster and delegated parenting to the middling sorts removing the condition of material necessity from the equation.^77^ Equally, the practice, openly advertised in the press, was framed as a form of delegated childcare and education when pursued by solvent mothers or families, as opposed to neglect, abandonment and an unnatural act, all terms used to describe such acts of poor mothers. Whilst those able to finance a *dignified* form of delegated childcare or pay for a discreet arrangement as offered by the two midwives were freed from unwanted parenthood, the same was not the case for the poor, who, unable to pay for their children’s maintenance, were themselves held responsible for the socially reproductive labours of parenting. Whilst notions of natural affection and blood ties as determinants of duty of care were invoked in the condemnation of poor mothers forsaking their children to infanticidal ‘baby farmers’, practices of abandonment, fostering and institutionalisation of unwanted children of the better-off were rarely portrayed in affective terms. Poor ‘unnatural’ mothers and ‘mercenary nurses’ were the characters evoked by those concerned for child welfare, whilst the better-off were rarely reprimanded for abandoning their unwanted children. Both Helen Laidlaw and Ann Alexander were involved in constructing childcare chains, with Alexander participating in them herself. Catering to the better off, both midwives’ services were sought by pregnant women for their ability to conceal their condition whilst providing care. The care work they offered was inextricable from its socio-cultural and economic contexts and their ability to adapt to these resulted in higher profit.

Offering care beyond childbirth, the Alexanders themselves provided the care that Mrs Laidlaw outsourced through a variety of channels. Midwifery was thus only an aspect of the complex forms of care and bodywork they performed, drawing on the common practice of boarding out children considered preferable to institutional care across Scotland.^78^ In spite of receiving somewhere between £13 and £30 annually for the care of a single child, the fostering practice of the Alexanders resembled that of the carers employed by the parish, paid significantly less. The monetary value of care was determined by who was doing the caring and for whom, with the physical and affective labours of care remaining constant. Reframed as a lucrative entrepreneurial pursuit as opposed to being the last resort of poor families in areas where little job prospects existed for married women, the Alexanders’ commodification of fostering again challenge the depiction of care as low paid and low status. Additionally, it contributes an important example of paid extra-familial care taking place within the domestic setting, posing challenges to the understanding of the family, household and parenting in early Victorian Scotland. Whilst Mrs Alexander’s familial, professional and residential circumstances changed, she continued to participate in a form of foster care throughout her life-cycle, with the practice providing her with a consistent source of income. Whilst no longer earning a significant sum through the practice, the physical, material and affective relations with the children she cared for would have remained unchanged, with the sum of money received unlikely playing a significant role in determining the relations that formed between the carer and the cared for.

## Conclusion

Apart from their reliance on the medium of newspaper advertising and providing care to pregnant women and their children, the lives and careers of Helen Laidlaw and Ann Alexander had little in common. Mrs Laidlaw, likely never-married, spent her life in the capital, running a prosperous private establishment, which allowed her to purchase real estate and expand her business. She worked as a midwife for over 50 years, frequently advertising in the popular press, which brought her to the attention of ‘pregnant women in need of retirement’ from across the British Isles. Her specialising in commercial care out with traditional networks of acquaintance and trust upon which most midwives relied, Mrs Laidlaw drew on Edinburgh’s nature as the medical capital, ensuring discretion to those who travelled to receive the treatment of private nature. Mrs Alexander worked for clients with similar needs, with Aberdeen providing a more remote alternative to Edinburgh. At the same time, however, Aberdeen lacked the network of practitioners and care providers who populated the capital and formed key channels of employment relations between medical men, midwives, nurses and casual care providers. Mrs Alexander’s practice was thus more isolated and the scope of her services even broader, perhaps a contributing factor to her business being short-lived. Unlike Helen Laidlaw, Ann Alexander ran her establishment alongside her husband, leaving the practice behind when the pair separated, though likely continuing as a midwife in a more localised setting of rural Aberdeenshire. Whilst it is probable that she continued her practice, no written evidence exists, highlighting once again the inadequacy of census records in documenting women’s working lives.^79^ In any case, working in textiles and fostering children charged on the parish in her old age, her midwifery practice cannot have been as prosperous. Unlike Mrs Laidlaw, Mrs Alexander’s work in the care sector was less stable, with the midwife resorting to various other forms of making a living alongside midwifery. In conjunction, the two midwives’ stories show the variety of work in the care sector, its opportunities for social mobility as well as its impermanence. Through their different life trajectories, the two women’s lives show the nature of care and bodywork as an opportunity for entrepreneurship, part of the makeshift economy, as well as a last resort, highlighting the diverse nature of commercial care.

Through the two case studies, this article first contributes to literature on late eighteenth and early nineteenth century midwifery, broadly contextualised by the history of women’s entrepreneurship. Despite the changing nature of healthcare over the course of the period, women continued to work as midwives, healthcare practitioners and bodyworkers across different settings. Some found avenues for entrepreneurship whilst others drew on the great demand for care work in the urban market in more casual ways. The commercial care sector was populated by a range of practitioners who pursued care work as a life-long occupation, acquiring training, qualifications and membership in professional bodies, as well as those who needed a temporary source of income. It formed a significant part of the expanding service sector, overlapping with domestic service, and lodging letting, washing and victualling amongst other forms of work. Drawing on the concept of bodywork, these varied forms of labour emerge as interconnected and interdependent, both paid and unpaid, casual as well as highly profitable. This article centred on two Scottish midwives and their diverse life trajectories. Their stories shed light on the complexity of care work, its at the same time public and private nature and its rootedness in the fabric of the urban economy. Highlighting the two women’s ability to capitalise on the moral economy of pregnancy and legitimacy, this article demonstrated the ways in which midwifery was at times practised outside of the localised networks of trust, operationalising the promise of secrecy to middling women unable or unwilling to take on the role of a mother. It highlighted the nature of care work beyond the underpaid and undervalued, instead demonstrating its variedness along the lines of gender, status and material circumstance that shaped the practices of care in the increasingly anonymised urban space.

## Footnotes

2
*Caledonian Mercury*, 19.5. 1832, 1.

3 Frances Badger, ‘Illuminating Nineteenth-Century English Urban Midwifery: The Register of a Coventry Midwife’, *Women’s History Review*, 2014, 23, 683–705, 5; Anne Cameron, ‘Female Birthing Customs and Beliefs’, in Deborah Simonton and Katie Barclay, eds, *Women in Eighteenth-Century Scotland: Intimate, Intellectual and Public Lives* (London: Routledge, 1991); Angela Joy Muir, ‘Midwifery and Maternity Care for Single Mothers in Eighteenth-Century Wales’, Social History of Medicine, 2018, 33, 394–416, 2.

4 T. C. Smout, *A History of the Scottish People* (Glasgow: HarperCollins Publishers Ltd., 1970); Michael Anderson, *Approaches to the History of the Western Family 1500-1914* (Cambridge: Cambridge University Press, 1980); Leah Leneman and Rosalind Mitchison, *Sin in the City: Sexuality and Social Control in Urban Scotland 1660-1780* (Edinburgh: Scottish Cultural Press, 1998); Leah Leneman and Rosalind Mitchison, *Girls in Trouble: Sexuality and Social Control in Rural Scotland, 1660-1780* (Edinburgh, 1998); Andrew Blaikie, *Illegitimacy, Sex and Society: Northeast Scotland, 1750-1900* (Oxford: Clarendon Press, 1994).

5 Hamish Mathison, ‘Tropes of Promotion and Wellbeing: Advertisement and the Eighteenth-Century Scottish Periodical Press’, *Prose Studies*, 1998, 21, 206–225, 2

6 See, for example: Amie Bolissian, ‘Masculine Old Women or Feminine Old Men? Rethinking Gender and the Ageing Body in Early Modern English Medicine’, *Gender & History*, 2023, 35, 408–28; Hannah Newton, *The Sick Child in Early Modern England, 1580-1720* (Oxford: Oxford University Press, 2012); Alexandra Shepard, ‘Care’, in C. Macleod, A. Shepard and M. Ågren, eds, *The Whole Economy: Work and Gender in Early Modern Europe* (Cambridge: Cambridge University Pres, 2023).

7 Mary Fissell, ‘Introduction: Women, Health and Healing in Early Modern Europe’, *Bulletin of the History of Medicine*, 2008, 82, 1–17; see also: Montserrat Cabré, ‘From a Master to a Laywoman: a Feminine Manual of Self-help’, *Dynamis: Acta Hispanica ad Medicinae Scientiarumque Historiam Illustrandam*, 2000, 20, 371–393; Sandra Cavallo, *Artisans of the Body in Early Modern Italy: Identities, Families and Masculinities* (Manchester: Manchester University Press, 2007).

8 Julia Twigg, Carol Wolkowitz, Rachel Lara Cohen, Sarah Nettleton, ‘Conceptualising Body Work in Health and Social Care’, *Sociology of Health and Illness*, 2011, 33, 171, 2; the authors exclude unpaid care from their definition, whilst my conceptualisation of bodywork includes both paid and unpaid care.

9
*Ibid.*

10 Barbara E. Mortimer, ‘The Nurse in Edinburgh c.1760-1860: The impact of commerce and professionalisation’ (Unpublished PhD thesis, University of Edinburgh, 2001); Barbara Mortimer, *New Directions in Nursing History* (London: Routledge, 2004); Barbara Mortimer.

11 For the ‘medical marketplace’, see: R. Colin Jones, ‘The Great Chain of Buying: Medical Advertisement, the Bourgeois Public Sphere, and the Origins of the French Revolution’, *The American Historical Review*, 1996, 1, 101; Roy Porter, *Health for Sale: Quackery in England 1660-1850* (Manchester: Manchester University Press, 1989); R. Porter, *Disease, Medicine and Society in England, 1550–1860,* second edition (Cambridge: Cambridge University Press, 1993); R. Porter and D. Porter, *In Sickness and in Health: The British Experience, 1650-1850* (London: Fourth Estate, 1988); R. Porter and J Brewer, *Consumption and the World of Goods* (London: Routledge, 1994); R. Porter and L. Grenshaw, *The Hospital in History* (London: Routledge, 1989); for broader concepts of the heterogeneity of practice see: Margaret Pelling, *The Common Lot, Sickness, Medical Occupations and the Urban Poor in Early Modern England* (New York: Routledge, 1998); Mark Jenner and Patrick Wallis, Introduction to: *Medicine and the Market in England and its Colonies, c.1450- c.1850* (Basingstoke: Palgrave Macmillan, 2007); Mary Fissell, ‘Women, Health and Healing in Early Modern Europe’, *Bulletin of the History of Medicine*, 2008, 82, 1; Mary Fissell, ‘Remaking the Maternal Body’, *Journal of the History of Sexuality*, 2017, 26, 1; Mary Fissell, ‘The Marketplace of Print’, in Mark Jenner and Patrick Wallis, *Medicine and the Market in England and its Colonies, c.1450- c.1850* (Basingstoke: Palgrave Macmillan, 2007).

12 On the man-midwife see: Adrian Wilson, ‘Midwifery in the “Medical Marketplace”’, in Jenner and Wallis, *Medicine and the Market in England and its Colonies, c.1450- c.1850* (Basingstoke: Palgrave and Macmillan, 2007); Adrian Wilson, *Ritual and Conflict: The Social Relations of Childbirth in Early modern England* (London: Routledge, 2013); Adrian Wilson, *The Making of Man-Midwifery* (Cambridge: Harvard University Press, 1995); Barbara Brandon Schnorrenberg ‘Is Childbirth Any place for a Woman? The Decline of Midwifery in Eighteenth-Century England’, *Studies in Eighteenth-Century Culture*, 1981, 10, 393–408; on midwifery more broadly: Jean Towler and Joan Bramall, *Midwives in History and Society* (London: Routledge, 1986); Leigh Whaley, *Women and the Practice of Medical Care in Early Modern Europe* (London: Palgrave McMillan, 2012); Lisa Forman Cody, ‘Mothers, Midwives and Mysteries’, in *Birthing the Nation: Sex, Science, and the Conception of Eighteenth Century Britons* (Oxford, Oxford University Press, 2005); Anne Borsay and Billie Hunter, eds, *Nursing and Midwifery in Britain since 1700* (Basingstoke: Palgrave Macmillan, 2012); on commercial care work: Mortimer, ‘The Nurse in Edinburgh’; on community practitioners: Elizabeth Ritchie, ‘The Township, the Pregnant Girl and the Church: Community Dynamics, Gender and Social Control in Early Nineteenth-Century Scotland’, *Northern Scotland,* 2019, 10, 41–67, 1; Elizabeth Ritchie, ‘‘‘A Palmful of Water for your Years’’ Babies, Religion and Gender Identity among Crofting Families, 1800-1850’, in Jodi A. Campbell, Elizabeth Ewan and Heather Parker, eds, *The Shaping of Scottish Identities: Family, Nation and the Worlds Beyond* (Guelph: Centre for Scottish Studies, 2011), 59–75.

13 For scholarship on male midwifery and the gendered conflict, see ft. 14; Ritchie, ‘The Township, the Pregnant Girl and the Church’.

14 Barbara Mortimer, ‘The Nurse in Edinburgh’; Anne Summers, ‘The Mysterious Demise of Sarah Gamp: The Domiciliary Nurse and Her Detractors, c. 1830-1860’, *Victorian Studies*, 1989, 32, 3; Badger, ‘Illuminating Nineteenth-Century English Urban Midwifery’.

15 See ft. 12 for literature on the ‘medical marketplace’ thesis and how it has been challenged.

16 Fissell, ‘The Marketplace of Print’.

17 Hannah Barker, *Newspapers and English Society, 1695-1855* (London: Routledge, 2000); Dingwall, ‘To be Insert in the Mercury’, 24; Mathison, ‘Tropes of Promotion and Wellbeing’.

18
*Caledonian Mercury*, 7.9. 1805, 1.

19 See, for example: *Morning Chronicle*, 2. 2. 1801, 1; *Caledonian Mercury*, 7.9. 1805, 1; *Caledonian Mercury*, 27.7. 1800, 1; *Sun (London)*, 5.3.1814, 4.

20
*Caledonian Mercury*, 7.9. 1805, 1.

21
*Morning Chronicle*, 2. 2. 1801, 1.

22 Jennifer Johnson-Hanks, ‘On the Limits of Life Stages in Ethnography: Toward a Theory of Vital Conjunctures’, *American Anthropologist*, 2002, 104, 865–880, 2.

23 Kate Gibson, *Illegitimacy, Family & Stigma in England, 1660-1834*, (Oxford: Oxford University Press, 2023); Linda A. Pollock, *Forgotten Children: Parent-Child Relations from 1500 to 1900* (Cambridge: Cambridge University Press, 1983); Patricia Crawford, *Parents of Poor Children in England 1580-1800* (Oxford: Oxford University Press, 2010); Katie Barclay, ‘Love, Care and the Illegitimate Child in Eighteenth-Century Scotland’, *Transactions of the Royal Historical Society*, 2019, 29, 105–125.

24 Laura Gowing, *Common Bodies, Women, Touch and Power in Seventeenth-Century England* (New Haven: Yale University Press, 2003); Muir, ‘Midwifery and Maternity Care’.

25 Katie Barclay, ‘Sex, Identity, and Enlightenment in the Long Eighteenth Century’, in Jodi A. Campbell, Elizabeth Ewan and Heather Parker, eds, *The Shaping of Scottish Identities: Family, Nation and the Worlds Beyond* (Guelph: Centre for Scottish Studies, 2011); Gibson, *Illegitimacy.*

26 Muir, ‘Midwifery and Maternity Care’.

27 Williamson’s directory for the City of Edinburgh, Canongate, Leith and suburbs, 1773-1776, 1784-1785, Mrs Laidlaw.

28
*Star (London),* 17.2.1814, 4; *Sun (London)*, 5.3.1814, 4.

29
*Ibid.*

30 Amy L. Erickson ‘Mistress and Marriage: or, a Short History of the Mrs’, *History Workshop Journal*, 2014, 78, 39–57.

31 Muir, ‘Midwifery and Maternity Care’; Tania McIntosh, ‘Profession, Skill, or Domestic Duty? Midwifery in Sheffield, 1881-1936’, *Social History of Medicine*, 1998, 11, 402–420, 3.

32 See ft. 12.

33 Edinburgh Dean of Guild Court, Box 1800/18, 19.6.1800, Thomson and Hutchison against Helen Laidlaw.

34 See, for example: *Caledonian Mercury*, 4.7. 1774, 3; *Caledonian Mercury*, 8.3. 1800, 1.

35 GUSC, GB 247 MS Hunter 500 (V.8.3), Lying-in Hospital Book, No. 1, 17.

36 Edinburgh Dean of Guild Court, Box 1800/18, 19.6.1800, Thomson and Hutchison against Helen Laidlaw.

37
*Ibid.*

38
*Morning Chronicle*, 2. 2. 1801, 1.

39
*Ibid.*

40
*Northampton Mercury*, 17.8.1793, 4; *Hereford Journal*, 7.12.1791, 2; *Bath Chronicle and Weekly Gazette*, 7.1.1808, 4.

41 See, for example: *Caledonian Mercury*, 7.9. 1805, 1; *Caledonian Mercury,* 19.5. 1805, 1.

42 Williamson’s directory for the City of Edinburgh, Canongate, Leith and suburbs, 1816, Mrs Laidlaw; Advertisement, Caledonian Mercury, 27th July 1809, 1, Advertisement, Caledonian Mercury, 12th October 1820, 1.

43
*Newcastle Courant*, 9. 7. 1803, 3; *Aberdeen Press and Journal*, 24. 7. 1816; *Caledonian Mercury*, 29.3. 1817, 1; Laidlaw vs Aldie, NRS CS36/18/50.

44 Laidlaw vs Aldie, NRS CS36/18/50.

45
*Caledonian Mercury*, 5.9. 1807, 3.

46
*Newcastle Courant*, 17.4. 1830, 1; *Caledonian Mercury*, 29.3. 1817, 1.

47 Mortimer, ‘The Nurse in Edinburgh’.

48
*Newcastle Courant*, 9.7. 1803, 3.

49
*Caledonian Mercury*, 5.9. 1807, 3.

50
*Caledonian Mercury*, 12.10. 1820, 1.

51
*Ibid.*

52 Laidlaw vs Blyth, NRS CS271/67674.

53 Gibson, *Illegitimacy.*

54
*Caledonian Mercury*, 12.10. 1820, 1.

55
*Perthshire Courier*, 17.8. 1815, 1.

56
*Cumbernauld Pacquet, and Ware’s Whitehaven Advertiser*, 12.2. 1828, 1.

57 McIntosh, ‘Profession, Skill, or Domestic Duty?’.

58 Cameron, ‘Female Birthing Customs and Beliefs’; Gowing, *Common Bodies.*

59
*The Scotsman*, 9.6.1841, 1.

60 I was able to find 18 adverts by Mrs Alexander or Mrs Alexander alongside William.

61 Cunynghame, ‘The Role of the Edinburgh, Kelso, and Newcastle Dispensaries’, 68.

62 See, for example: Amy L. Erickson, ‘Married women’s occupations in eighteenth-century London’ *Continuity and Change*, 2008, 23, 2; Maria Ågren, *Making a Living, Making a Difference: Gender and Work in Early Modern European Society*, (Oxford: Oxford University Press, 2017).

63 Alison Nuttall, ‘Midwifery, 1800-1920, The Journey to Registration’, in Anne Borsay and Billie Hunter, eds, *Nursing and Midwifery in Britain since 1700* (Basingstoke: Palgrave Macmillan, 2012); see also: Badger, ‘Illuminating Nineteenth-Century English Urban Midwifery’.

64 For women’s entrepreneurship, see: Erickson, ‘Married women’s occupations’; Eliska Bujokova, ‘On the respectability of this person every thing depends’: hospital matrons and power relations in the Royal Infirmary of Edinburgh, c. 1817–1820’, *Women’s History Review*, 2023, 32, 5; Catriona M. MacLeod, ‘Women, Work and Enterprise in Glasgow, c.1740-1830’, (Unpublished PhD Thesis, University of Glasgow, Faculty of History, 2015); Peter Earle, ‘The Female Labour Market in London in the Late Seventeenth and Early Eighteenth Centuries’, *The Economic History Review, New Series*, 1989, 42, 328–53, 3; Ågren, *Making a Living.*

65
*Aberdeen Press and Journal*, 9.2. 1848, 1; *Aberdeen Press and Journal*, 22.9.1847, 4.

66
*Aberdeen Press and Journal*, 9.12.1846, 1.

67 In Edinburgh, 21 midwives recorded in 1820 and 78 in 1837, compared with 99 surgeons in 1820 and 217 in 1837. In Aberdeen, 24 midwives recorded in 1829 and 47 in 1843, compared with 26 and 48 surgeons in the respective years. Williamson’s directory for the City of Edinburgh, Canongate, Leith and suburbs, 1820, 1837; Scottish Post Office Directory, Aberdeen, 1829, 1843.

68 Valerie Fildes, *Wet Nursing, A History from Antiquity to the Present* (Edinburgh: Edinburgh University Press, 1988); Ruth E. Homrighaus, ‘Wolves in Women’s Clothing: Baby-farming and the British Medical Journal, 1860-1872’, *Journal of Family History*, 2001, 26, 350–1, 3.

69 Jonas Hanway, An Earnest Appeal for Mercy to the Children of the Poor (1766).

70 ‘Baby-Farming’, *British Medical Journal*, 19 October 1867.

71 See for practices of fostering in Scotland: Janay Nugent, ‘‘Your louing childe and foster’: The Fostering of Archie Campbell of Argyll, 1633-39’, in Janay Nugent and Elizabeth Ewan, eds, *Children and Youth in Premodern Scotland* (London: Boydell and Brewer, 2015); Helen J. MacDonald, ‘Boarding-out and the Scottish Poor Law, 1845-1914’, *Scottish Historical Review*, 1996, 75, 197–220, 200; Philip Seed, ‘Should any Child be Placed in Care? The Forgotten Great Debate 1841-74’, *British Journal of Social Work*, 1973, 3, 321–330, 3; see also current research undertaken by Kate Gibson, ‘Looked-after Children: Fostering and Adoption in Britain, 1700-1839’ (University of Manchester, Leverhulme ECF).

72
*Bell’s Life in London and Sporting Chronicle*, 4.7.1841, 1.

73 Carmen Sarasúa, Pilar Erdozáin, Ricardo Hernández, ‘Nursing babies to fight poverty: Wages of Wet Nurses of Spanish Foundling Hospitals in the 18th and 19th Centuries’, *Journal of Iberian and Latin American Economic History*, 2023, 42, 1–29.

74 See ft. 80.

75 ‘Glasgow Parochial Board Report’ in *Glasgow Herald*, 14.8.1848, 4.

76
*The Scotsman*, 11.9. 1844, 4.

77 Nugent, ‘Your louing childe and foster’.

78 See ft. 80.

79 Badger, ‘Illuminating Nineteenth-Century English Urban Midwifery’.

## Notes

**Eliska Bujokova** is a researcher at the Glasgow University Centre for Gender History focussing on the histories of care, institutional welfare and gender and work. Her research published in Women’s History Review and Continuity and Change has focussed on women’s work in the care sector and the developments of Scottish systems of poor relief. Her interests broadly span labour, social, economic and medical histories across the British Atlantic with a particular focus on gender.

